# Arteriovenous fistulas in hemodialysis: factors of success and the role of nephrologists

**DOI:** 10.1590/2175-8239-JBN-2018-0161

**Published:** 2018-12-17

**Authors:** Ricardo Portiolli Franco

**Affiliations:** 1 Fundação Pro-Renal Brasil, Curitiba, PR, Brasil.

Arteriovenous fistulas are considered the access of choice for hemodialysis (HD) due to the lower incidence of complications, morbidity, and costs in relation to grafts and catheters.

The benefits of fistulas led to the creation of the Fistula First Initiative, which reduced graft rates in the United States after the year 2000. However, these efforts also increased the rates of maturation failure, now recognized as the main obstacle to achieve a functioning fistula. The need for angioplasty procedures to aid the maturation of many of these accesses also increased^1^.

**Figure 1:**
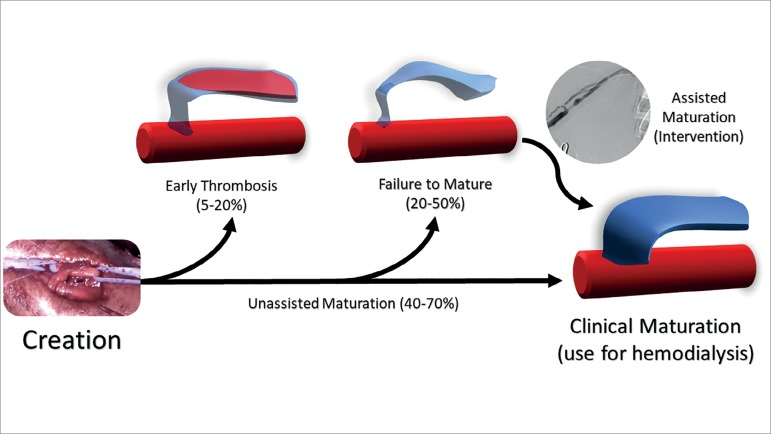
Possible outcomes after fistula creation. Early thrombosis occurs in up to 20% of cases and disrupts the development of the fistula. Failure to mature is usually caused by venous stenosis. In these cases endovascular interventions may promote maturation. Up to 50% of fistulas reach maturation without intervention.

The clinical maturation of a fistula can be achieved in up to 80% of the cases, however up to half of the fistulas need some intervention. To help with fistula maturation there are potential therapeutic targets. In the preoperative period, mapping with ultrasonography for evaluation of the venous and arterial diameters allows the selection of the best access site. Virtually all cases of maturation failure present stenosis, which can be treated successfully in up to 90% of cases^2^. The endovascular treatment of the stenosis increases the chance of maturation, increasing the possibility of salvage in cases of thrombosis. Figure 1 summarizes the possible outcomes of the fistulas after their creation.

In this issue of the BJN, Rodrigues et al. reports on a series of retrospective cases, studying two aspects of fistula creation: variables associated with the immediate and late success of arteriovenous fistulas and fistula creation by nephrologists^3^.

A total of 159 fistulas were created in 101 patients and the immediate patency, defined as presence of thrill or pulse in the immediate postoperative period, and late patency, defined as the possibility of use after 30 days, were evaluated. Considering the factors related to the patient, intraoperative high blood pressure (> 140/90 mmHg), Ca x P < 55, and hemoglobin between 10 and 12 mg/dL correlated with immediate patency and only hemoglobin levels correlated with late patency. Other variables such as sex, age, presence or absence of diabetes, and location of the fistula (proximal or distal) did not correlate with patency rates.

These findings add to the present divergence regarding factors related to maturation failure. The literature is conflicting about demographic and clinical factors such as female gender, age, presence of diabetes, and coronary artery disease. It is believed that these factors are more important when they directly affect hemodynamic factors that act in maturation, the main one being the diameter of the vessels used. The minimum diameter is 2.5 mm for veins and 2 mm for arteries.

Recently, the Haemodialysis Fistula Maturation Consortium prospectively evaluated several aspects of fistula maturation. The evaluation included 602 participants in pre-dialysis follow-up, of which 44% achieved unassisted maturation, 27%, assisted maturation, and 22% failed to mature. The evaluation of early thrombosis, in up to 18 days, found the variables female gender, localization in the forearm, venous diameter less than 2 to 3 mm, and use of protamine to be related positively with the maturation failure^4^. A second evaluation studied the correlation of intimal hyperplasia and venous stenosis in pre- and post-operative evaluation with failure to mature^5^. Only postoperative venous stenosis correlated with maturation failure and its effect could not be distinguished from other indicators, such as flow and venous diameter at 6 weeks. The findings corroborate the importance of factors that directly affect the hemodynamics of the fistula in its maturation.

The second point discussed by Rodrigues et al. is the safety and efficacy of fistula created by nephrologists. Interventional nephrology and its participation in endovascular procedures are well documented realities, proving that these specialists can perform the procedure with safety and efficacy. The author achieved 78% of immediate patency and 69.2% of late patency. The complication rate was 3.6%. These results are comparable with those of the international literature. Mishler evaluated the literature on the participation of nephrologists in the creation of accesses in 8 countries, showing outcomes consistent with other specialists. It is interesting to cite a Spanish service that obtained a reduction in the average waiting time from 103 to 25 days and a reduction in the use of catheters in incident patients from 63 to 19% 6. Early access to the nephrologist and the initiation of HD with a fistula reduces mortality, according to a Brazilian report by Diegoli et al^7^.

In Japan, nephrologists participate in approximately 30% of endovascular treatments, fistula creation, and prostheses. According to a personal report of Dr. Masanobu Gunji, nephrologist of the Mito Saisekai General Hospital, in his service, 95% of the accesses are created by him. The waiting time for fistulas is at most one day and his clinic has zero patient using central venous catheters. According to Dr. Gunji, vascular surgeons do the complex cases and there are case discussions among the specialties. In his own words, "we cooperate".

The field of vascular accesses is multidisciplinary and requires interaction between different specialties. The report by Rodrigues et al. reinforces that with specific training nephrologists can successfully create arteriovenous fistulas with low complication rates. This does not limit the participation nor the need of vascular surgeons dedicated to the management of HD accesses, especially in more complex cases. Considering the resources available in each service, the participation of trained nephrologists has the potential to reduce waiting times and the time of central venous catheters exposure.
